# Efficacy and safety of Kami-guibi-tang for mild cognitive impairment: a pilot, randomized, double-blind, placebo-controlled trial

**DOI:** 10.1186/s12906-021-03428-6

**Published:** 2021-10-07

**Authors:** Hee-Yeon Shin, Ha-Ri Kim, Geon-Ho Jahng, Chul Jin, Seungwon Kwon, Seung-Yeon Cho, Seong-Uk Park, Woo-Sang Jung, Sang-Kwan Moon, Chang-Nam Ko, Jung-Mi Park

**Affiliations:** 1 Department of Clinical Korean Medicine, Graduate School, Kyung Hee University, 26, Kyungheedae-ro, Dongdaemun-gu, Seoul, 02447 Republic of Korea; 2 Department of Radiology, Kyung Hee University Hospital at Gangdong, College of Medicine, Kyung Hee University, 892, Dongnam-ro, Gangdong-gu, Seoul, 05278 Republic of Korea; 3 Department of Cardiology and Neurology, College of Korean Medicine, Kyung Hee University, 26, Kyungheedae-ro, Dongdaemun-gu, Seoul, 02447 Republic of Korea; 4 Stroke and Neurological Disorders Center, Kyung Hee University Hospital at Gangdong, 892, Dongnam-ro, Gangdong-gu, Seoul, 05278 Republic of Korea

**Keywords:** Mild cognitive impairment, Amnestic mild cognitive impairment, Herbal medicine, Traditional Korean medicine, Kami-guibi-tang, Seoul neuropsychological screening battery, Clinical dementia rating

## Abstract

**Background:**

Mild cognitive impairment (MCI) is considered an intermediate phase between normal aging and dementia. As the majority of cases of amnestic MCI (aMCI) progress to Alzheimer’s disease (AD), it is considered the prodromal stage of AD, and a treatment target for prevention of further cognitive decline. However, no medications have been shown to have symptomatic or preventive benefits in MCI. Kami-guibi-tang (KGT) is a traditional herbal formula used in Korean medicine to treat amnesia, which is reported to increase acetylcholine levels via activation of choline acetyltransferase. The objective of this study was to evaluate the efficacy and safety of KGT in patients with aMCI.

**Methods:**

This study was designed as a single-center, randomized, double-blind, placebo-controlled pilot study. Participants diagnosed with aMCI were randomized to receive either KGT or placebo granules for 24 weeks. The efficacy measure was a change in the Seoul Neuropsychological Screening Battery (SNSB) score. The safety measures included the occurrence of adverse events and abnormalities in vital signs and blood chemistry, electrocardiogram (ECG), and brain magnetic resonance imaging (MRI) findings.

**Results:**

A total of 16 patients in the KGT group and 14 patients in the placebo group were investigated in the study. The mean score of Clinical Dementia Rating-Sum of Boxes (CDR-SB) significantly improved from 1.53 (0.64) points to 1.13 (0.62) points in the KGT group (*p* = 0.010), whereas it worsened from 1.61 (0.88) points to 1.75 (0.94) points in the placebo group. There was a significant difference in the CDR-SB scores between the two groups after the intervention (*p* = 0.045). The total SNSB-D scores and the scores in the memory domain after the treatment were significantly higher than the baseline values in the KGT group, but not in the placebo group. The frequency of adverse events was not significantly different between the two groups, and there were no abnormalities in vital signs or blood test, ECG, and brain MRI findings after the intervention.

**Conclusions:**

KGT may provide a safe and effective treatment option for patients with aMCI. Further studies with a larger sample size are needed to validate the findings.

**Trial registration:**

Korean Clinical Trial Registry, ID: KCT0002407; Registered on March 30, 2017, http://cris.nih.go.kr/

**Supplementary Information:**

The online version contains supplementary material available at 10.1186/s12906-021-03428-6.

## Background

Mild cognitive impairment (MCI) is the intermediate stage between the cognitive changes of normal aging and dementia [1]. It is a syndrome defined as a cognitive decline greater than that expected for an individual’s age and educational level but that does not interfere with the performance of activities of daily living (ADLs) [2]. The incidence of MCI in individuals aged over 70 years was estimated to be 5 to 6% per year [3].

Amnestic MCI (aMCI), which is the most common subtype of MCI, is characterized by memory impairment [4] and usually progresses to Alzheimer’s disease (AD) [5]. Therefore, it is often considered the prodromal stage of AD [6]. It is reported that 10 to 15% of patients with aMCI develop AD per year, while 1 to 2% of healthy subjects develop AD per year [7].

As the prevalence of AD increases with advancing age [8], interest in the early treatment of MCI has been stimulated by the hope that intervention at this stage may delay the progression to AD [9]. A variety of pharmaceutical agents have been tested, including cholinesterase inhibitors (ChEIs), which are the standard for symptomatic treatment of AD [10, 11]; however, there is currently no medication proven to enhance memory function or prevent further cognitive decline in patients with MCI [12, 13].

Kami-guibi-tang (KGT; Kami-guibi-tang in Korean, Kami-kihi-to in Japanese, Jia-wei-gui-pi-tang in Chinese) is a traditional herbal formula that has been used to treat amnesia [14], insomnia [15], loss of appetite [16] for hundreds of years in Korea, Japan and China [17–19]. KGT improved deficits in object recognition memory in 5XFAD mice, a transgenic animal model of AD, which exhibit accelerated amyloid-beta deposition [20]; it also improved learning performance in a mouse model of accelerated senescence [21]. Moreover, a clinical study showed that the Mini Mental State Examination (MMSE) score of patients with AD improved after taking oral kihito granules [22]. A cross-over clinical study revealed that the MMSE score significantly increased during the kihito intake period but not during the ChEI alone treatment period [23].

As shown in previous studies [20–23], KGT was reported to have a beneficial effect on cognitive improvement in patients with AD. We hypothesized that KGT might also enhance cognitive function, specifically memory function, in aMCI patients. To our knowledge, no randomized clinical trial has been conducted to evaluate the effect of KGT in MCI. This pilot study aimed to evaluate the efficacy and safety of KGT in improving cognitive function of patients with aMCI.

## Methods

### Study design

The study protocol has been previously published [24]. This study was designed as a single-center, randomized, double-blind, placebo-controlled, parallel-group clinical trial. It was conducted at Kyung Hee University Hospital at Gangdong, Seoul, Korea from March 2017 to November 2019. The study consisted of three phases: a screening period lasting 2 weeks, a treatment period lasting 24 weeks, and a follow-up period for adverse events lasting 4 weeks.

The protocol of this trial was approved by the Institutional Review Board of Kyung Hee University Hospital at Gangdong (KHNMC-OH-IRB 2016–12–006-006) and the Korean Ministry of Food and Drug Safety (31234), and was registered at the Korean Clinical Trial Registry (Registration number: KCT0002407, http://cris.nih.go.kr/). All participants provided voluntary signed informed consent before participating in this study. The study subjects were limited to amnestic subtype of MCI, which has preserved abilities in other cognitive functions and independent performances of ADLs. Also, the study investigators assessed their decisional capacity through interviews and neuropsychological test. The participants were considered capable of giving consent for participation in research, and therefore, it was regarded unnecessary to use surrogate consent.

This trial was conducted in accordance with the Declaration of Helsinki principles and the Korean Good Clinical Practice guidelines.

### Participants

#### Inclusion criteria

Participants were included in the study if they were aged between 55 and 90 years old; complained of impaired memory; had objective cognitive impairment as measured using the Seoul Neuropsychological Screening Battery (SNSB), with a Global Deterioration Scale (GDS) score of 3 points, clinical dementia rating (CDR) of 0.5 points, and a normal Korean MMSE (K-MMSE) score; and were diagnosed with aMCI by a neurologist.

#### Exclusion criteria

Participants were excluded if they met any of the following criteria: diagnosed with AD according to the criteria of the National Institute of Neurological and Communicative Disorders and Stroke and Alzheimer’s Disease and Related Disorders Association; had a brain disorder causing neurological symptoms other than cognitive impairment; a diagnosis of Parkinson’s disease, Huntington’s disease, Down’s syndrome, Creutzfeldt-Jakob disease, or any other neurodegenerative disorder; cognitive impairment resulting from other diseases, including head trauma, hypoxic brain damage, vitamin deficiency, brain tumor, encephalitis, neurosyphilis, and mental retardation; cerebrovascular diseases with magnetic resonance imaging (MRI) evidence; a history of or current major depression; concomitant psychiatric disorders or behavioral problems that require antipsychotic medication; a history of a convulsive disorder, except for febrile convulsion during childhood; unstable or life-threatening medical conditions; uncontrolled hypertension; heart or renal diseases; peripheral edema; gastrointestinal symptoms, such as anorexia, nausea, abdominal pain, or diarrhea; use of medications that could induce hypokalemia or myopathy; drug hypersensitivity to the constituents of the study medication; clinically significant abnormalities in blood chemistry test results, including levels of serum aspartate aminotransferase (AST)/alanine aminotransferase (ALT) more than two-fold the upper normal limit or serum creatinine (Cr) level more than 10% above the upper normal limit; participation in any other clinical trials within the previous 4 weeks; illiteracy; and contraindications for MRI.

#### Dropout criteria

Participants who met any of the following criteria were removed from the study: (1) occurrence of any severe adverse effects, (2) voluntary withdrawal from the trial, (3) non-observance of the protocol (i.e., drug compliance below 80%), (4) use of additional medications to improve cognitive function during the study period, and (5) decision made by the principal investigator.

#### Recruitment and enrollment

We recruited participants aged 55–90 years with a complaint of impaired memory through advertisements and referrals. We screened candidates using telephone interviews, the inclusion/exclusion criteria, and K-MMSE and Korean Dementia Screening Questionnaire (KDSQ) findings. If the score of delayed recall items on the K-MMSE is less than 2 points out of 3, the SNSB was performed. If the score of any subtests in the memory domain of SNSB was less than 16 percentile, and if a neurologist confirmed the diagnosis of aMCI, the participant was included in the trial.

#### Randomization and blinding

We randomly allocated the enrolled participants to either the treatment (KGT) or control (placebo) group in a 1:1 ratio, using the block randomization method with a block size of four. A researcher uninvolved in the assessment generated a random sequence using IBM SPSS Statistics 20.0 (IBM Co., Armonk, NY, USA). The manufacturer produced KGT and placebo granules to be identical in appearance, smell, taste, and packaging, and labeled the random code numbers on the medication kit. An independent pharmacist distributed the granules to the participants in the order of enrollment. The participants, assessor, pharmacist, and researchers were all blinded to the allocation throughout the course of the study. Cases were unblinded only if serious adverse events occurred.

## Intervention

After randomization, the KGT group received KGT granules (3.0 g/pack), and the placebo group received placebo granules (3 g/pack). The participants were instructed to dissolve the granules in hot water and drink the solution three times per day, 30 min after meals. KGT, the herbal medication under study, is a yellow-brown mixture of spray-dried hot water extracts of 15 medicinal herbs. The composition and amount of each ingredient are shown in Table 1. The KGT granules used were manufactured by Kyoung Bang Pharmaceutical Co., Ltd. (Incheon, Korea), which has been certified for Good Manufacturing Practice (GMP). The placebo granules were composed of corn starch, lactose, hydroxypropyl cellulose, caramel color (food additives), tartrazine (FD&C Yellow 5), Allura Red AC (FD&C Red 40), and Ssanghwa flavor. They were produced by the same manufacturer, using the standard method of placebo manufacturing according to the Korean GMP guidelines.

**Table 1:** Composition of Kami-guibi-tang

Scientific name	Latin name	Amount (g)
*Panax ginseng C.A. Meyer*	Ginseng Radix	1
*Atractylodes macrocephala Koidzumi*	Atractylodis Rhizoma Alba	1
*Poria cocos Wolf*	Poria Sclerotium	1
*Astragalus membranaceus Bunge*	Astragali Radix	1
*Dimocarpus longan Loureiro*	Longanae Arillus	1
*Zizyphus jujuba Miller var. spinosa* *Hu ex H.F. Chou*	Zizyphi Semen	1
*Bupleurum falcatum Linné*	Bupleuri Radix	1
*Angelica gigas Nakai*	Angelicae Gigantis Radix	0.67
*Polygala tenuifolia Willdenow*	Polygalae Radix	0.67
*Gardenia jasminoides Ellis*	Gardeniae Fructus	0.67
*Paeonia suffruticosa Andrews*	Moutan Cortex	0.67
*Zizyphus jujuba Miller var. inermis Rehder*	Zizyphi Fructus	0.67
*Aucklandia lappa Decne*	Aucklandiae Radix	0.33
*Glycyrrhiza uralensis Fischer*	Glycyrrhizae Radix et Rhizoma	0.33
*Zingiber officinale Roscoe*	Zingiberis Rhizoma Recens	0.33

* *Dimocarpus longan* used in this trial was cultivated for medicinal purposes, and not harvested from the wild

The participants were provided with 12 weeks’ worth of trial drugs at the first and second visits. They were required to return any unused drugs at the second and third visits. The number of returned drugs was counted to evaluate drug compliance, and participants with less than 80% compliance were excluded.

The administration of medications for underlying diseases, such as hypertension or diabetes mellitus, was permitted during the intervention; however, any medication that might affect cognitive function was prohibited. We asked participants to report all medications taken during the study period at each visit and recorded the names, duration of use, and dosage of the drugs on the case report form.

## Assessments

### Korean ini-mental state examination and Seoul neuropsychological screening battery

The K-MMSE was performed at the screening visit and on the 12th week of intervention to rule out normal cognition and dementia, and to screen for MCI. It is a simple screening test for the longitudinal assessment of general cognition [25].

The SNSB was conducted by an independent clinical psychologist at baseline and the 24th week of intervention to evaluate the effect of KGT on cognitive function. It is a valid and reliable neuropsychological test battery for assessing and monitoring cognitive function, which is one the most widely used assessments in South Korea [26]. The SNSB-II, a revised version of the SNSB, is composed of subtests that evaluate five cognitive aspects: attention, language, memory, visuospatial function, and executive function [27].

The SNSB test yields scores, percentile ranks in a graph and a table. 50%ile means the average of individuals at the same age or educational level. It is regarded that there is cognitive impairment if the score is less than 16%ile (1 standard deviation [SD]). If the memory domain is less than 16%ile, it was considered there was objective memory impairment.

A modified version of the original SNSB for dementia (SNSB-D) was used to assess the general cognitive function using a score drawn from the sum of the score for the five domains [28]. The maximum total score is 300 points consisting of 17/300 points (6%) for attention, 27/300 points (9%) for language and related function, 150/300 points (50%) for memory, 36/300 points (12%) for visuospatial function, and 70/300 points (23%) for frontal/executive function. The contents of the SNSB-D are shown in Additional File 2.

Other related tests, such as the K-MMSE, CDR scale, GDS, Barthel-Activities of Daily Living (Barthel-ADL) scale, Korean-Instrumental Activities of Daily Living (K-IADL) scale, and Short version-Geriatric Depression Scale (SGDS), were also conducted (Additional File 3).

The CDR scale and GDS are the most well-known rating scales that measure the symptoms and severity of dementia [29]. The CDR scale yields a global score (CDR-GS) and a sum of boxes score (CDR-SB). The CDR-GS is used to stage dementia severity on a 5-point scale that ranges between 0 and 3, where 0 = absence of symptoms, 0.5 = questionable, 1 = mild, 2 = moderate, and 3 = severe dementia. The CDR-SB, which refers to the sum of the score for six domains (three cognitive domains: orientation, memory, and judgment and problem-solving; three functional domains: home and hobbies, personal care, and community affairs), is used to measure global cognition and functioning [30]. A higher score indicates more severe impairment. The GDS scale is used to assess the stages of cognitive decline on a 7-point scale [31].

Two instruments were used to assess ADLs. The Barthel-ADL scale measures the ability to perform ADLs, such as feeding, bathing, and dressing [32]. The K-IADL scale is used to evaluate instrumental ADLs, which are more complex activities, such as using the telephone, preparing food, and taking medications [33]. The SGDS is a screening tool for depression in elderly patients [34].

#### Blood test and electrocardiogram (ECG)

Laboratory tests and ECGs were performed to evaluate the safety of KGT. Blood levels of blood urea nitrogen, Cr, AST, ALT, Na, K, Cl, creatine phosphokinase, lactate dehydrogenase, and glucose were measured at baseline and on the 12th and 24th weeks of intervention. ECG was performed at baseline and the 24th week.

#### Brain MRI

Brain MRI was performed at baseline and the 24th week. Three-dimensional T1-weighted images, two-dimensional T2-weighted images, and fluid attenuation inversion recovery sequences were acquired to evaluate structural abnormalities in the brain.

### Outcome variables

#### Efficacy outcome

We observed the changes in the mean SNSB-D score and other indices, including the CDR, GDS score, K-MMSE score, Barthel-ADL scale score, K-IADL scale score, and SGDS score, before and after administration of the KGT and placebo granules and compared the changes between the KGT and placebo groups. In particular, the change in general cognition was assessed on the basis of the change in the total SNSB-D score and CDR-SB score. The change in each cognitive domain was assessed on the basis of the changes in the five SNSB-D subtest scores.

#### Safety outcome

The safety of KGT was evaluated by monitoring any occurrence of adverse events and any abnormalities in the vital signs and blood test, ECG, and brain MRI findings. The investigators assessed any adverse events face to face at each visit and via telephone interviews every week during the intervention period and 4 weeks after the completion of the study. The severity and relationship of the adverse events to the study drug were assessed.

### Statistical analysis

#### Sample size calculation

This trial was a pilot study, and we could not find previous data indicating the sample size needed to yield significant results for determining the effect of KGT as assessed using the SNSB scores. For pilot trials, a sample size of 20–40 participants was suggested by Kieser and Wassmer [35]. Furthermore, Browne suggested a general flat rule to use at least 30 subjects to estimate a parameter [36]. Therefore, a sample size of 30 participants was determined to be adequate, and we attempted to recruit 38 individuals at maximum to allow for a 20% dropout rate.

#### Data analyses

All the data acquired from the study were entered into Microsoft Excel 2010 (Microsoft Co., Redmond, *WA*, USA), and the statistical analysis was performed by the investigators and an independent professional statistician using IBM SPSS Statistics 20.0 (IBM Co.).

The analysis of treatment efficacy was performed on the per-protocol (PP) set. The PP population included participants who completed taking the study medication for 24 weeks and underwent all examinations. The analysis of the treatment safety was performed on the intention-to-treat (ITT) set. The ITT population included those who took the KGT and placebo granules more than once after randomization.

To analyze the efficacy outcome and demographic variables, such as age and educational level, we performed the Shapiro-Wilk test to confirm the normality of data distribution. For normally distributed data, the paired t-test was used for intra-group comparisons and independent t-test for intergroup comparisons. For non-normally distributed data, Wilcoxon’s signed rank test was used for intra-group comparisons and the Mann-Whitney U test for intergroup comparisons. To analyze the K-MMSE score, which was measured three times, the Friedman test was performed. To compare baseline variables, such as sex, conversion to normal cognition or dementia, and occurrence of adverse events, between groups, we conducted Pearson’s chi square test or Fisher’s exact test, as appropriate.

All statistical analyses were two-tailed, and the significance level was set at *p*-values of < 0.05. Continuous variables are presented as means (standard deviations) or medians (interquartile ranges) and categorical variables as numbers (percentages).

## Results

### Participants

**Fig. 1:**
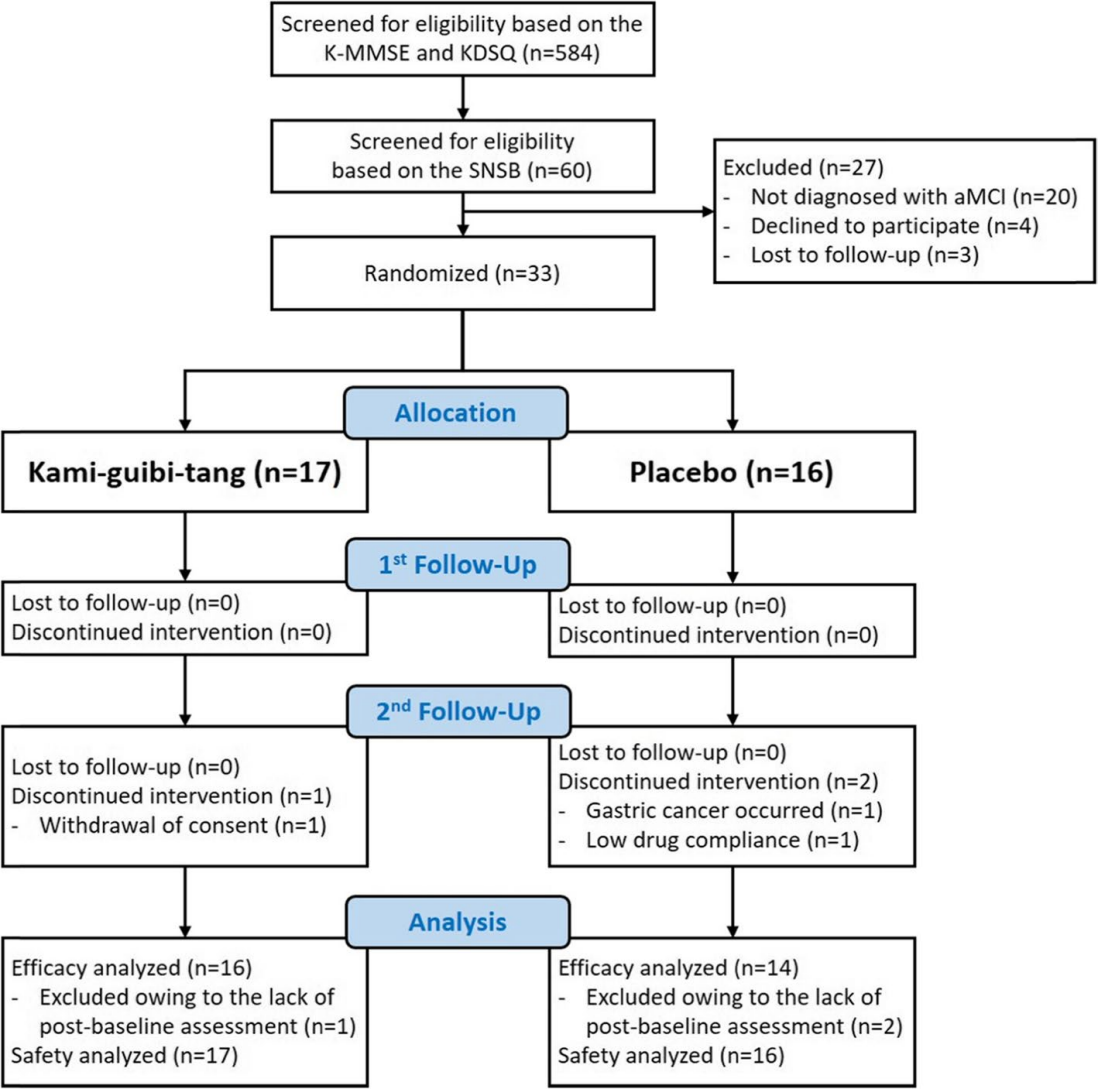
CONSORT diagram. Abbreviation: K-MMSE, Korean Mini Mental Status Examination; KDSQ, Korean Dementia Screening Questionnaire; SNSB, Seoul Neuropsychological Screening Battery; aMCI, amnestic mild cognitive impairment

We recruited participants from June 2017 to March 2019. A total of 584 participants were screened via telephone interviews; on the basis of the K-MMSE and KDSQ findings, there were 60 participants with suspected MCI. After evaluation using the SNSB test, a total of 33 participants who were finally diagnosed with aMCI were enrolled. Seventeen participants were assigned to the KGT group and 16 participants to the placebo group. The details of the recruitment, enrollment, and distribution of the participants are demonstrated in Fig. 1. All 33 randomized participants were included in the safety analysis. Sixteen participants from the KGT group and 14 from the placebo group were included in the efficacy analysis (Fig. 1).

No significant differences were observed in the demographic variables or efficacy outcomes, such as the baseline SNSB-D score, CDR, GDS score, K-MMSE score, Barthel-ADL score, K-IADL score, and SGDS score (Table 2), between the groups following randomization.

**Table 2:** Demographic and baseline characteristics

	KGT group (*n* = 16)	Placebo group (*n* = 14)	p-value^*^
Demographics			
Age	70.2 (7.6)	70.1 (6.4)	0.986
Sex (Male)	10 (62.5%)	7 (50%)	0.491
Educational level	12.2 (3.6)	12.3 (4.7)	0.967
Clinical characteristics			
SNSB-D	176.00 (24.76)	173.79 (32.86)	0.835
Attention	9.5 (2.3)	9.3 (1.3)	0.783
Language and related function	23.6 (2.3)	22.1 (3.3)	0.232
Visuospatial function	32.94 (2.82)	33.18 (3.05)	0.732
Memory	57.88 (17.76)	57.39 (17.97)	0.942
Frontal/executive function	52.1 (9.2)	51.8 (12.8)	0.946
Other indices			
CDR-SB	1.50 (1.00–2.00)	1.50 (0.88–2.50)	0.793
CDR-GS (0.5)	16 (100%)	14 (100%)	–
GDS (3)	16 (100%)	14 (100%)	–
K-MMSE	28.5 (26.3–29.0)	26.0 (24.8–28.3)	0.067
Barthel-ADL	20	20	–
K-IADL	1.0 (1.0–2.0)	1.0 (1.0–1.3)	0.510
SGDS	4.0 (1.3–10.8)	2.5 (1.8–9.0)	0.645

KGT, Kami-guibi-tang; SNSB-D, Seoul Neuropsychological Screening Battery for dementia; CDR-SB, Clinical Dementia Rating-Sum of Boxes; CDR-GS, Clinical Dementia Rating-Global Score; GDS, Global Deterioration Scale; K-MMSE, Korean Mini Mental State Examination; SGDS, Short version-Geriatric Depression Scale; K-IADL, Korean-Instrumental Activities of Daily Living; Barthel-ADL, Barthel-Activities of Daily Living

Values are presented as means (standard deviations), medians (Q1–Q3), or numbers (%)

^*^p-values for the between-group comparison using the independent t-test or chi-square test (or Fisher’s exact test)

### Efficacy outcome

#### Seoul neuropsychological screening battery for dementia (SNSB-D)

In the KGT group, the total SNSB-D score increased significantly from 176.00 (24.76) points at baseline to 198.47 (31.29) points at the 24th week (*p* < 0.001, Table 3); however, the change was not significant in the placebo group (*p* = 0.143, Table 3). There was no significant difference in the SNSB-D score at the 24th week between the groups (*p* = 0.249, Table 3).

**Table 3:** SNSB-D scores at baseline and the 24th week

Variables	KGT group (n = 16)			Placebo group (n = 14)			Between-group comparison
	Baseline	24th week	p-value^†^	Baseline	24th week	p-value^†^	p-value^*^
Total SNSB-D score	176.00 (24.76)	198.47 (31.29)	< 0.001	173.79 (32.86)	182.14 (44.25)	0.143	0.249
Attention	10.0 (8.0–11.0)	11.0 (7.3–12.0)	0.285	9.5 (8.0–10.3)	10.0 (7.8–11.0)	0.418	0.541
Language and related function	23.5 (22.3–25.0)	24.5 (22.0–26.0)	0.510	23.0 (20.0–24.3)	23.5 (21.8–25.0)	0.510	0.264
Visuospatial function	34.00 (30.25–35.00)	34.00 (32.25–35.00)	0.304	34.50 (31.75–35.00)	33.50 (32.00–35.00)	1.000	0.622
Memory	57.88 (17.76)	74.34 (22.66)	< 0.001	57.39 (17.97)	66.25 (28.94)	0.051	0.398
Frontal/executive function	52.1 (9.2)	56.4 (8.4)	0.127	51.8 (12.8)	50.4 (12.3)	0.462	0.125

SNSB-D, Seoul Neuropsychological Screening Battery for dementia; KGT, Kami-guibi-tang

Values are presented as means (standard deviations) or medians (Q1–Q3)

^†^*p*-value for the intergroup comparison using the paired t-test or Wilcoxon’s signed rank test

^*^p-value for the between-group comparison of the 24th-week scores using the independent t-test or Mann-Whitney U test

Among the scores for the five cognitive domains, the score for the memory domain in the KGT group increased significantly from 57.88 (17.76) points at baseline to 74.34 (22.66) points at the 24th week (p < 0.001, Table 3); however, the change was not significant in the placebo group (*p* = 0.051, Table 3). There was no significant difference in the score for the memory domain at the 24th week between the groups (*p* = 0.398, Table 3).

Among the scores for the memory domain subsets, the Seoul Verbal Learning Test (SVLT) recall test score increased from 17.5 (15.0–25.3) points to 24.5 (15.5–29.8) points; the Rey Complex Figrue Test (RCFT) recall test score increased from 20.00 (10.30) points to 29.91 (13.54) points; and the RCFT recognition score increased from 5.9 (2.6) points to 7.6 (1.9) points after 24 weeks of intervention in the KGT group (*p* = 0.003, *p* < 0.001, and *p* = 0.002, Table 4); however, the changes in the placebo group were not significant (*p* = 0.081, *p* = 0.062, and *p* = 0.223, respectively, Table 4). There was no significant difference in the scores at the 24th week between the two groups (*p* = 0.190, *p* = 0.660, and *p* = 0.821, respectively, Table 4).

**Table 4:** Memory domain subset scores at baseline and the 24th week

Variables	KGT group (*n* = 16)			Placebo group (*n* = 14)			Between-group comparison
	Baseline	24th week	p-value^†^	Baseline	24th week	p-value^†^	p-value^*^
Memory	57.88 (17.76)	74.34 (22.66)	< 0.001	57.39 (17.97)	66.25 (28.94)	0.051	0.398
Orientation	6.0 (6.0–6.0)	6.0 (5.3–6.0)	0.257	6.0 (5.0–6.0)	6.0 (5.0–6.0)	0.564	0.340
SVLT recall	17.5 (15.0–25.3)	24.5 (15.5–29.8)	0.003	17.0 (13.0–21.3)	20.0 (13.0–26.3)	0.081	0.190
SVLT recognition	7.0 (6.0–8.0)	7.5 (5.3–10.0)	0.109	6.0 (4.0–8.0)	6.0 (3.8–8.3)	0.826	0.158
RCFT recall	20.00 (10.30)	29.91 (13.54)	< 0.001	20.89 (10.43)	27.32 (18.25)	0.062	0.660
RCFT recognition	5.9 (2.6)	7.6 (1.9)	0.002	6.6 (2.1)	7.4 (2.8)	0.223	0.821

KGT, kami-guibi-tang; SVLT, Seoul Verbal Learning Test; RCFT, Rey Complex Figure Test

Values are presented as means (standard deviations) or medians (Q1–Q3)

^†^p-value for the intergroup comparison using the paired t-test or Wilcoxon’s signed rank test

^*^p-value for the between-group comparison of the 24th-week scores using the independent t-test or Mann-Whitney U test

### Other cognitive tests

#### Clinical dementia rating-sum of boxes (CDR-SB)

The CDR-SB score improved significantly from 1.53 (0.64) points to 1.13 (0.62) points after the intervention in the KGT group (*p* = 0.010, Table 5); however, it worsened from 1.61 (0.88) points to 1.75 (0.94) points in the placebo group. There was a significant difference in the score at the 24th week between the two groups (*p* = 0.045, Table 5).

**Table 5:** CDR-SB scores at baseline and the 24th week

Variables	KGT group (n = 16)			Placebo group (n = 14)			Between-group comparison
	Baseline	24th week	p-value^†^	Baseline	24th week	p-value^†^	p-value^*^
CDR-SB score	1.53 (0.64) 1.50 (1.00–2.00)	1.13 (0.62) 1.00 (0.50–1.50)	0.010	1.61 (0.88) 1.50 (0.88–2.50)	1.75 (0.94) 1.75 (0.88–2.50)	0.453	0.045

CDR-SB, Clinical Dementia Rating-Sum of Boxes; KGT, Kami-guibi-tang

Values are presented as means (standard deviations) and medians (Q1–Q3)

^†^p-value for the intergroup comparison using Wilcoxon’s signed rank test

^*^p-value for the between-group comparison of the 24th-week scores using the Mann-Whitney U test

#### Clinical dementia rating-global score (CDR-GS)

At baseline, all participants had a CDR-GS score of 0.5 point, which indicates “questionable dementia.” After 24 weeks of intervention, 31.25% of the KGT group participants had a reversion of the CDR-GS score to 0 point, which indicates “no dementia,” in comparison with 14.29% of the placebo group participants. The reversion rate was not significantly different between the two groups (*p* = 0.399, Table 6). The CDR-GS score did not increase to 1 point, which indicates “mild dementia,” in any participant.

**Table 6:** Reversion rate to normal cognition as measured using the CDR-GS score after 24 weeks of intervention

CDR	KGT group (n = 16)	Placebo group (n = 14)	p-value^‡^
Reversion to normal (CDR of 0.5 points → 0 points)	5 (31.25%)	2 (14.29%)	0.399
Stable MCI (CDR of 0.5 points)	11 (68.75%)	12 (85.71%)	
Conversion to dementia (CDR of 0.5 points → ≥1 point)	0 (0%)	0 (0%)	

CDR-GS, Clinical Dementia Rating-Global Score; KGT, Kami-guibi-tang; MCI, Mild cognitive impairment

Values are presented as numbers (%)

^‡^p-value for the between-group comparison using Fisher’s exact test

#### Other indices

The K-MMSE, GDS, K-IADL scale, and SGDS scores did not change significantly after the intervention in both groups, and there were no significant differences in the scores at the 24th week between the two groups (Table 7).

**Table 7:** K-MMSE and other test scores at baseline and the 12th and 24th week

Variables	KGT group (n = 16)				Placebo group (n = 14)				Between-group comparison
	Baseline	12th week	24th week	p-value^§^	Baseline	12th week	24th week	p-value^§^	p-value^*^
K-MMSE score	28.5 (26.3–29.0)	28.5 (27.0–30.0)	28.0 (26.3–29.8)	0.168	26.0 (24.8–28.3)	26.5 (24.5–28.0)	27.5 (25.0–28.0)	0.667	0.097
GDS									
2	0 (0%)		5 (31.25%)	–	0 (0%)		2 (14.29%)	–	0.399
3	16 (100%)		11 (68.75%)	–	14 (100%)		12 (85.71%)	–	
Barthel-ADL	20		20	–	20		20	–	–
K-IADL	1.0 (1.0–2.0)		1.0 (1.0–1.0)	0.438	1.0 (1.0–1.3)		1.0 (1.0–2.0)	1.000	0.994
SGDS	4.0 (1.3–10.8)		2.0 (1.0–8.5)	0.063	2.5 (1.8–9.0)		4.5 (1.0–8.3)	0.591	0.464

K-MMSE, Korean Mini Mental State Examination; KGT, Kami-guibi-tang; GDS, Global Deterioration Scale; SGDS, Short version-Geriatric Depression Scale; K-IADL, Korean-Instrumental Activities of Daily Living; Barthel-ADL, Barthel-Activities of Daily Living

Data are presented as medians (Q1–Q3) or numbers (%)

^§^p-value for the intergroup comparison using the Friedman test

^*^p-value for the between-group comparison using the Mann-Whitney U test or Pearson’s chi-square test

### Safety outcome

#### Adverse events

Approximately 11.8% of the KGT group participants and 12.5% of the placebo group participants reported adverse events. There was no significant difference in the occurrence of adverse events between the two groups (*p* = 1.0).

In the KGT group, one participant had temporomandibular joint pain and one had mild dyspepsia. These symptoms appeared after taking the medication for more than 12 weeks and subsided spontaneously within 2 weeks. In the placebo group, one patient had heartburn and another developed gastric cancer. There was no link found between these adverse events and the study drug.

#### Examinations

There were no significant changes in the vital signs, ECG, blood test including BUN, Cr, AST, ALT, Na, K, Cl, CPK, LDH, and glucose, or brain MRI findings after the intervention in both groups. There were no significant abnormalities observed in any of the above-mentioned examinations.

## Discussion

In this study, the general cognition of participants as measured using the CDR-SB score improved significantly in the KGT group unlike those in the placebo group; further, general cognition as measured using the total SNSB-D score improved significantly in the KGT group in comparison with their baseline score. Their memory as measured using the SNSB-D subtest scores improved significantly in the KGT group after the intervention.

In previous studies, the MMSE has been mainly used to measure cognitive function; however, it lacks sensitivity in detecting mild degrees of cognitive dysfunction and demonstrates ceiling effects in MCI [37]. Other instruments, including the Alzheimer’s Disease Assessment Scale-cognitive subscale, often lack the ability to assess certain cognitive domains, such as attention and executive function. In this study, we used the full neuropsychological battery, SNSB, which contains comprehensive and diverse tests of varying difficulties, to sensitively monitor changes in cognitive function [26].

The SNSB-D is a useful tool that can discriminate between MCI, AD, and normal aging [28]. A study reported that the change in the SNSB-D score was significantly correlated with the percent change in the cortical gray matter volume in an aMCI group; therefore, the SNSB-D score is capable of estimating neurodegenerative changes [37]. Our finding that the SNSB-D score increased significantly indicates that KGT might not only improve cognitive symptoms but may also influence disease progression.

Among the five cognitive domains, the memory domain was the most remarkably affected domain. Memory impairment is a core feature of aMCI and AD [38, 39]. We observed that both verbal (SVLT recall) and visual memory (RCFT recall and RCFT recognition) improved after taking the KGT granules. This is particularly notable considering that verbal memory impairment is the best neuropsychological predictor of further cognitive decline and progression to AD [40].

Other studies using the CDR-SB showed that there was no significant difference in the change in the CDR-SB score between the rofecoxib and placebo groups [41] and that it worsened less in the galantamine group than in the placebo group [11]. This trial showed that the CDR-SB scores differed significantly between the two groups after the intervention. Moreover, the CDR-SB score decreased in the KGT group but increased in the placebo group, which means that KGT granules improved global cognition and functioning.

We also attempted to estimate how KGT might influence disease progression from the CDR-GS score. It was reported that the annual reversion rate from MCI to normal cognition was 18.20% [42]. In this study, the rate was 14.29% in the placebo group compared to 31.25% in the KGT group, which was higher than the natural course. Although there was no significant difference, KGT might delay the disease progression, considering the significant increase in the CDR-SB score.

There was no significant improvement in the scores for other cognitive domains, except for memory. This may be because the study population was limited patients with aMCI, whose cognitive function in other domains is relatively preserved. Among the other related tests included in the SNSB-II, there was no significant improvement, except for in the CDR. It is presumable that the inclusion of only participants with normal MMSE and ADL scores in this trial has contributed to the outcome. Further, we excluded individuals with a history of depression to eliminate any bias from cognitive decline caused by depression, which might have resulted in the high average SGDS score.

Although the exact mechanism by which KGT ameliorates cognitive decline is unknown, possible mechanisms have been suggested in previous studies. Among the 15 medicinal herbs of KGT, *Polygalae Radix* is believed to play a key role in improving cognitive function, by elevating acetylcholine levels via activation of choline acetyltransferase [43]. It is similar to ChEIs, which inhibit cholinesterase, in that both increase the level of acetylcholine; however *P. radix* is known to have additional neuroprotective effects by enhancing the axonal length after A*β*-induced axonal atrophy and inhibiting neural damage [44].

In addition to improving cognitive symptoms, the beneficial effect of KGT on AD pathology has been demonstrated in several studies. It is reported that KGT not only reduced the number of amyloid plaques in the frontal cortex and hippocampus [20] but also reversed the progression of A*β*-induced axonal degeneration in cortical neurons. KGT treatment also led to tau dephosphorylation in primary cultured cortical neurons that were phosphorylated by A*β* treatment [45] . These results indicate the possible mechanisms explaining how KGT might slow the disease progression of aMCI.

This trial showed that KGT was safe and well tolerated, as noted in previous studies [46, 47]. The frequency of adverse events did not differ significantly between the KGT and placebo groups. The adverse events that occurred in the KGT group were mild and transient and disappeared spontaneously without the need for discontinuing medication. As ChEIs often cause side effects, including gastrointestinal symptoms and cardiac concerns, the safety of KGT could be another advantage.

There are several limitations in this study. First, the sample size was relatively small; however, the sample size was enough for a pilot study to achieve the goal of evaluating the efficacy of KGT. Second, there was no long-term follow-up examination. As MCI can progress slowly over time, it is necessary to monitor the effect over a long-term basis. Third, the study excluded patients with depression, although a majority of patients with MCI exhibited neuropsychological symptoms. As KGT can be also used for insomnia, anorexia, and depression, further studies should include patients with MCI and concurrent psychological symptoms. However, considering the impact that treating depression may have on cognitive function, this study aimed to differentiate the effect of KGT on improving cognitive function from the effects secondary to mood improvement.

In this study, we strengthened the assessment so that we could identify specifically how much cognitive function improved. Further, we also attempted to remove the learning effect from repetitive measurement by setting 24 weeks as the period between the baseline and endpoint measurements. The study is also the first clinical study that investigated the effects of KGT on aMCI. We hope that our findings will support a future large-scale clinical trial to generate confirmatory evidence for the use of KGT in patients with aMCI.

## Conclusions

This pilot study aimed to explorethe efficacy and safety of KGT for aMCI. The trial demonstrated that KGT may be effective in improving general cognition in patients with aMCI and that it was generally safe and well tolerated. The findings highlight the potential of KGT as a possible treatment option for aMCI. Larger-scale clinical trials are needed to substantiate clinical evidence on the benefits of KGT and to identify the mechanisms underlying its efficacy.

## Supplementary Information

**Additional File 1.** CONSORT 2010 Checklist

**Additional File 2.** Table S1. Construction of the SNSB-D

**Additional File 3.** Table S2. Other indexes included in the SNSB-II

## Data Availability

The datasets used and/or analyzed during the current study are available from the corresponding author on reasonable request.

## Abbreviations

MCI: mild cognitive impairment
aMCI: amnestic mild cognitive impairment
AD: Alzheimer’s disease
KGT: Kami-guibi-tang
SNSB: Seoul Neuropsychological Screening Battery
ECG: electrocardiogram
MRI: magnetic resonance imaging
SVLT: Seoul verbal learning test
ChEI: cholinesterase inhibitor
MMSE: Mini Mental State Examination
GDS: Global Deterioration Scale
CDR: clinical dementia rating
K-MMSE: Korean Mini Mental Status Examination
AST: aspartate aminotransferase
ALT: alanine aminotransferase
Cr: creatinine
KDSQ: Korean Dementia Screening Questionnaire
GMP: Good Manufacturing Practice
SNSB-D: Seoul Neuropsychological Screening Battery for dementia
SVLT: Seoul Verbal Learning Test
RCFT: Rey Complex Figure Test
Barthel-ADL: Barthel-Activities of Daily Living
K-IADL: Korean-Instrumental Activities of Daily Living
ADL: activities of daily living
SGDS: Short version-Geriatric Depression Scale
CDR-GS: clinical dementia rating-global score
CDR-SB: clinical dementia rating-sum of boxes
PP: per-protocol
ITT: intention-to-treat
