# Supplementary material for: Efficacy and safety of Kami-guibi-tang for mild cognitive impairment: a pilot, randomized, double-blind, placebo-controlled trial
Source: BMC Complement Med Ther. 2021 Oct 7;21:251. doi: 10.1186/s12906-021-03428-6 (PMC8495912; doi:10.1186/s12906-021-03428-6)
Supplement: Supplementary file 2 — Additional File 2. Table S1. Construction of the SNSB-D [file 12906_2021_3428_MOESM2_ESM.docx]

| Table S1. The construction of SNSB-D | | |
| --- | --- | --- |
| **Domains** | **Subtests** | **Scores** |
| **Attention (17)** | Digit span forward | / 9 |
|  | Digit span backward | / 8 |
| **Language &**  **Related Function (27)** | K-BNT | / 15 |
|  | Calculation | / 12 |
| **Visuospatial Function (36)** | Rey copy | / 36 |
| **Memory (150)** | Orientation | / 6 |
|  | SVLT recall | / 48 |
|  | SVLT recognition | / 12 |
|  | Rey recall | / 72 |
|  | Rey recognition | / 12 |
| **Frontal & Executive**  **Function (70)** | Impersistence | / 3 |
|  | Contrasting program | / 3 |
|  | Go-no-go test | / 3 |
|  | Fist-edge-palm | / 3 |
|  | Luria loop | / 3 |
|  | Word fluency : animal | / 20 |
|  | Word fluency : korean | / 15 |
|  | Stroop test | / 20 |
| **Total SNSB-D** |  | **/ 300** |
|  | | |
