# Supplementary material for: Efficacy and safety of Kami-guibi-tang for mild cognitive impairment: a pilot, randomized, double-blind, placebo-controlled trial
Source: BMC Complement Med Ther. 2021 Oct 7;21:251. doi: 10.1186/s12906-021-03428-6 (PMC8495912; doi:10.1186/s12906-021-03428-6)
Supplement: Supplementary file 3 — Additional File 3. Table S2. Other indexes included in the SNSB-II [file 12906_2021_3428_MOESM3_ESM.docx]

| Table S2. Other indexes included in SNSB-II | | |
| --- | --- | --- |
| **Cognitive tests** | | **Scores** |
| **CDR** | Clinical Dementia Rating Scale |  |
|  | CDR-GS: Global Score | 0~5 |
|  | CDR-SB: Sum of Boxes | / 18 |
| **GDS** | Global Deterioration Scale | 1~7 |
| **K-MMSE** | Korean-Mini Mental State Examination | / 30 |
| **Barthel-ADL** | Barthel-Activities of Daily Living | / 20 |
| **K-IADL** | Korean-Instrumental Activities of Daily Living |  |
| **SGDS** | Short Version of Geriatric Depression Scale | / 15 |
|  | | |
